# Role of Exendin-4 in Brain Insulin Resistance, Mitochondrial Function, and Neurite Outgrowth in Neurons under Palmitic Acid-Induced Oxidative Stress

**DOI:** 10.3390/antiox10010078

**Published:** 2021-01-09

**Authors:** Danbi Jo, Gwangho Yoon, Juhyun Song

**Affiliations:** 1 Department of Anatomy, Chonnam National University Medical School, Hwasun 58128, Korea; 198390@jnu.ac.kr (D.J.); ghyoon@chonnam.ac.kr (G.Y.); 2 BK21 PLUS Center for Creative Biomedical Scientists at Chonnam National University, Research Institute of Medical Sciences, Chonnam National University Medical School, Gwangju 501-757, Korea

**Keywords:** exendin-4 (Ex-4), glucagon like petide-1 (GLP-1), neuronal structure, obesity, palmitic acid (PA)

## Abstract

Glucagon like peptide 1 (GLP-1) is an incretin hormone produced by the gut and brain, and is currently being used as a therapeutic drug for type 2 diabetes and obesity, suggesting that it regulates abnormal appetite patterns, and ameliorates impaired glucose metabolism. Many researchers have demonstrated that GLP-1 agonists and GLP-1 receptor agonists exert neuroprotective effects against brain damage. Palmitic acid (PA) is a saturated fatty acid, and increases the risk of neuroinflammation, lipotoxicity, impaired glucose metabolism, and cognitive decline. In this study, we investigated whether or not Exentin-4 (Ex-4; GLP-1 agonist) inhibits higher production of reactive oxygen species (ROS) in an SH-SY5Y neuronal cell line under PA-induced apoptosis conditions. Moreover, pre-treatment with Ex-4 in SH-SY5Y neuronal cells prevents neural apoptosis and mitochondrial dysfunction through several cellular signal pathways. In addition, insulin sensitivity in neurons is improved by Ex-4 treatment under PA-induced insulin resistance. Additionally, our imaging data showed that neuronal morphology is improved by EX-4 treatment, in spite of PA-induced neuronal damage. Furthermore, we identified that Ex-4 inhibits neuronal damage and enhanced neural complexity, such as neurite length, secondary branches, and number of neurites from soma in PA-treated SH-SY5Y. We observed that Ex-4 significantly increases neural complexity, dendritic spine morphogenesis, and development in PA treated primary cortical neurons. Hence, we suggest that GLP-1 administration may be a crucial therapeutic solution for improving neuropathology in the obese brain.

## 1. Introduction

Metabolic syndrome is a major global health issue, and is gradually increasing in prevalence worldwide, at all ages [1]. Obesity is one of these metabolic syndromes, and is rapidly growing in incidence; obesity is accompanied by dyslipidemia, hypertension, diabetes, and cognitive dysfunction [2]. Epidemiological studies have presented a strong relationship between obesity and memory loss in humans [3,4]. Hyperglycemia in obesity causes vascular damage, brain atrophy, and impaired neurogenesis in the brain [5]. It has also been shown to increase the risk of Alzheimer’s disease (AD) by promoting severe neuroinflammation, insulin resistance, synaptic dysfunction, and impairments in cerebral vasculature [6]. Moreover, obese patients showed brain insulin resistance, increased neuroinflammation response, and excessive saturated fatty acids, such as palmitate, both in circulation and the cerebrospinal fluid [7,8].

The consumption of high-fat diets with saturated fatty acids, such as palmitic acid (PA), leads to an increase in weight, and triggers metabolic impairment, such as insulin resistance [9,10]. High PA levels can trigger endoplasmic reticulum (ER) stress, cell death, and impaired differentiation in neurons [11,12]. PA-induced neuronal cell damage induces an AD-like pathological pattern in primary cortical neurons (PCNs) [13]; a decrease of proliferation in neural progenitor cells [14] and neural cell apoptosis [15]. Furthermore, recent studies have reported that a high accumulation of PA in neurons caused by a high-fat diet induces brain insulin resistance and impaired long-term potentiation; and this leads to memory dysfunction [16] and an increased production of amyloid-beta in neuronal cells [17].

Despite the evidence showing that excessive PA levels in the brain are harmful to memory function and increase the risk of neurodegenerative diseases, an appropriate therapy for neuronal protection against PA-induced damage is lacking.

Glucagon like peptide 1 (GLP-1) is an incretin hormone secreted by L cells in the gut, and by neuronal cells from the tractus solitarius of the brain stem [18]. GLP-1 controls glucose metabolism and insulin sensitivity [19] by binding to GLP-1 receptors in diverse brain regions, including the hypothalamus, hippocampus, and cortex [20]. L-cell-derived GLP-1 may enter the brain through the blood [21]. GLP-1, and exendin-4 (Ex-4) and liraglutide (GLP-1 receptor agonists) inhibit oxidative stress-related neuronal loss, and improve synaptic plasticity and the neuronal network [22,23]. Considering that the enhancement of neuronal morphology and the neural network is strongly linked to the progress of neuronal disorders [24], GLP-1′s effect on neuronal connectivity is important for finding therapies for neurodegenerative diseases.

In this study, we investigated whether the GLP-1 agonist, Ex-4, improves neuronal connectivity and neuronal function in neurons under PA-induced oxidative stress. Our data demonstrates the potential of GLP-1 for the treatment of neuropathology in the obese brain.

## 2. Materials and Methods

### 2.1. Drug Treatments and Preparation

Ex-4 was purchased from Tocris Bioscience (Avonmouth, Bristol, UK), and diluted using sterilized water. PA and all-trans retinoic acid (RA) were purchased from Sigma Aldrich (St. Louis, MO, USA). RA was diluted using anhydrous dimethyl sulfoxide. PA was conjugated with bovine serum albumin (PA-BSA) in three steps. First, 1 g of PA powder was dissolved in 7.8 mL ethanol (99%) (500 mM solution) at 35–40 °C, and the solution was filtered using a 0.2 μm filter. Next, 1.5 g of BSA was diluted in 15 mL of serum-free media at 37 °C, and filtered using a 0.45 μm filter. Finally, we obtained 5 mM of PA-BSA by mixing the two solutions at a ratio of 100 (BSA solution): 1 (PA solution). Cells were treated with 10 nM Ex-4 for 2 h before treatment with 50 μM PA. To evaluate insulin signaling in PA-induced insulin resistance, cells were treated with 20 nM of insulin for 10 min, and subsequently collected for western blot analysis.

### 2.2. SH-SY5Y Cell Culture and Differentiation

The human neuroblastoma SH-SY5Y cell line (ATCC, Manassas, VA, USA) was cultured in minimum essential medium (MEM) with 10% fetal bovine serum (FBS), 100 U/mL penicillin-streptomycin, and 1 mM sodium pyruvate. Cells (4.0 × 10^4^ cells/cm^2^) were seeded in cell culture dishes or plates, and were cultured at 37 °C with 5% CO_2_. After 1 day of seeding, the medium was changed to MEM containing 2% FBS and 100 U/mL penicillin-streptomycin with 20 μM RA, for differentiation. The medium was replaced every 2 days; the cells were treated with 10 nM Ex-4 and 50 μM PA-BSA, as appropriate. Differentiation was performed for up to 4 days. The detailed culture schedule is described as (supplementary information Figure S1A,B).

### 2.3. Rat Primary Cortical Neuron Culture and Transfection

Rat PCNs were obtained from the cerebral cortices of Sprague-Dawley rat embryos (Koatech, Pyeongtaek, Korea) on embryonic day 14. The isolated cortices were rapidly collected in 1× Hank’s balanced salt solution, washed three times in a dissection/dissociation medium, and were incubated in 0.5% trypsin for 15 min at 37 °C. Subsequently, the cells were washed twice with the dissection/dissociation medium, and were seeded in a medium comprising MEM with Earle’s balanced salt solution, 0.45% glucose, 10% FBS, 1× GlutaMAX-Q, 1× sodium pyruvate, and 100 U/mL penicillin-streptomycin. Cortices were separated into single cells, and the cells (1.4 × 10^4^ cells/cm^2^) were seeded in poly-L-lysine-coated culture plates. For immunostaining, primary cortical cells were cultured on cover slips coated with poly-L-lysine, in culture plates. After 1 h the medium was changed to maintenance medium containing Neurobasal medium, 1× B-27, 1× GlutaMAX-Q, 100 U/mL penicillin-streptomycin, and 0.5 μM cytosine arabinoside. The maintenance medium was replaced every 3 days. Neurite complexity analysis, western blotting, and immunostaining experiments were performed on day 7 in vitro (DIV7), and dendritic spine analysis was performed on day 21 in vitro (DIV21).

To analyze neurite complexity and the dendritic spines of PCNs, cells were transfected with the pMAX-GFP plasmid (Lonza, Basel, Switzerland) using FuGENE 6 transfection reagent (Promega, Madison, WI, USA), according to the manufacturer’s instructions. After 24 h at 37 °C, neurite outgrowth and dendritic spines were analyzed in the cells. The detailed culture schedule is described in Figure S1C,D. All primary culture procedures were performed in accordance with the Animal Care Guidelines of Chonnam National University, South Korea.

### 2.4. Quantitative Real Time Polymerase Chain Reaction (PCR)

Total RNA was extracted using TRIzol reagent (Ambicon, Austin, TX, USA) according to the manufacturer instructions, and complementary DNA was reverse-transcribed from the extracted RNA using RevertAid reverse transcriptase (Thermo Fisher Scientific, Waltham, MA, USA). The complementary DNA was quantified using a NanoPhotometer (IMPLEN, Westlake village, CA, USA). Quantitative real time PCR was performed using 10 ng of complementary DNA with the Power SYBR green PCR master mix and the Step One Plus real time PCR system (Applied Biosystems, Foster City, CA, USA). The expression level of each gene was normalized to that of Glyceraldehyde 3-phosphate dehydrogenase (GAPDH). The primer information is described in Figure S1E.

### 2.5. Western Blot Analyses

Cells were lysed in radioimmunoprecipitation lysis buffer (Translab, Dajeon, Korea) containing protease inhibitors and phosphatase inhibitors. Protein concentrations were quantified using a BCA assay kit (Thermo Fisher Scientific) according to the manufacturer’s instructions. Protein (25 μg) was separated by 8–12% SDS-PAGE, and transferred onto a methanol-activated Polyvinylidene difluoride membrane (Millipore, Burlington, MA, USA). Membranes were blocked with 5% skim-milk or 5% BSA in 1× tris-buffered saline-tween (TBS-T) (10X TBS buffer, 0.1% Tween-20) for 1 h at room temperature (RT). Membranes were incubated with primary antibodies (1:1000 dilution) in 1× TBS-T containing 5% BSA, at 4 °C overnight. After primary antibody binding, membranes were incubated with secondary antibodies (horseradish peroxidase (HRP)-labeled mouse anti-rabbit IgG (Santa Cruz Biotechnology, Dallas, TX, USA) or goat anti-mouse IgG (Santa Cruz Biotechnology); 1:5000 dilution) in 1× TBS-T buffer for 2 h at RT. Membranes were treated with ECL solution (Thermo Fisher Scientific) and analyzed using Fusion Solo software (Vilber, Marne-la-Vallée, France). The protein expression level was measured using ImageJ software. All protein levels were normalized to β-actin levels. Phosphorylated protein levels were normalized to native form levels, and cleaved protein levels were normalized to full-length protein levels.

The primary antibodies used were as follows: BCL2-associated X, apoptosis regulator (Bax, #5023), B-cell lymphoma 2 (Bcl-2, #4223), cysteine-aspartic acid protease 3 (Caspase-3, #9662), β-actin, phosphatidylinositol kinase (PI3K)/protein kinase B (AKT, #4691), p-AKT (Ser473, #9271), phosphorylated glycogen synthase kinase 3 beta (p-GSK-3β, Ser9, #9323), peroxisome proliferator-activated receptor gamma (PPAR-γ, #2435), p44/42 MAPK extracellular signal-regulated kinases 1/2 (ERK1/2, #9102), p-ERK1/2 (Thr202/Tyr204, #4370), cAMP-response element binding protein (CREB, #9104), p-CREB (Ser133, #9198), and postsynaptic density protein 95 (PSD-95, #3409) (all from Cell signaling, Danvers, MA, USA). Cleaved-Poly (ADP-ribose) polymerase 1 (PARP-1, ab4830), peroxisome proliferator-activated receptor gamma coactivator 1-alpha (PGC-1α, ab54481), and microtubule-associated protein 2 (MAP-2, ab32454) antibodies were purchased from Abcam (Abcam, Cambridge, UK). Insulin receptor substrate 1 (IRS-1, PA1-1057) and p-IRS-1 (Tyr612, 44-816G), GSK-3β (sc-9116) and β-tubulin (sc-9935), and synaptophysin (SYP, MAB368) antibodies were purchased from Invitrogen (Invitrogen, San Diego, CA, USA), Santa Cruz Biotechnology, and Millipore (Millipore, Burlington, MA, USA), respectively.

### 2.6. Reactive Oxygen Species (ROS) Assay

SH-SY5Y cells were seeded into 96-well plates, and treated with PA (50 μM) and Ex-4 (10 nM) for 24 h. To estimate intracellular ROS under PA treatment, SH-SY5Y cells were incubated with 100 μM 2′,7′-Dichlorodihydrofluorescin diacetate for 1 h at RT. Cells were washed thrice with 1× phosphate-buffered saline (PBS). Fluorescence intensity was measured using a SpectraMax M2 microplate reader (Molecular Devices, San Jose, CA, USA), with excitation at 485 nm and emission at 520 nm.

### 2.7. Transferase dUTP Nick End Labeling (TUNEL) Staining

To measure the distribution of apoptosis in PA-treated SH-SY5Y cells, we used a DeadEnd Fluorometric terminal deoxynucleotidyl TUNEL system kit (Promega, Madison, WI, USA), according to the manufacturer’s instructions. Cells were imaged with an Eclipse Ts2 fluorescent microscope (Nikon, Tokyo, Japan), and TUNEL-positive cells were analyzed using ImageJ software.

### 2.8. Immunocytochemistry

SH-SY5Y cells (4 × 10^4^ cells/cm^2^) and PCNs (1.4 × 10^4^ cells/cm^2^) were seeded on coated cover slips in culture plates. Cells were fixed with 2% paraformaldehyde (PFA) for 10 min at RT. The PFA was discarded, and the cells were washed thrice with 1× PBS. They were then incubated with primary antibodies in GDB buffer (0.1% Gelatin, 0.3% Triton X-100, 16 mM sodium phosphate, pH 7.4, 450 mM NaCl) (1:200 dilution) at 4 °C overnight. Subsequently, the cells were washed thrice with 1× PBS, and incubated with GDB buffer containing the secondary antibodies (1:200 dilution) for 1 h at RT. Cell nuclei were counterstained using a mounting solution with 4′,6-diamidino-2-phenylindole (DAPI). Cells were visualized using a Zeiss LSM 710 confocal microscope (Carl Zeiss, Oberkochen, Germany). Fluorescence intensities were analyzed using ImageJ software.

Primary antibodies were used as the following antibodies: Cleaved-caspase3 (Cell signaling, #9664), p-IRS1 (Invitrogen, residues surrounding Tyr612, 44-816G), PGC-1α (Abcam, ab54481), PSD-95 (Cell signaling, #3409). Secondary antibodies used were as follows: secondary antibodies (horseradish peroxidase (HRP)-labeled mouse anti-rabbit IgG (Santa Cruz Biotechnology, sc2357).

### 2.9. Mitochondrial Activity Analyses

To analyze mitochondrial activity, cells were stained with 200 nM MitoTracker Deep Red FM (Invitrogen, San Diego, CA, USA) for 30 min. Subsequently, the staining solutions were removed, and the cells were washed with 1× PBS, fixed with 2% PFA for 15 min, and counterstained using a mounting solution with DAPI. Mitochondrial activity was imaged using a Zeiss LSM 710 confocal microscope (Carl Zeiss, Oberkochen, Germany), and analyzed using ImageJ software.

### 2.10. Neurite Outgrowth and Viability Measurement

SH-SY5Y cells were stained with the neurite outgrowth staining kit (Life Technologies, Carlsbad, CA, USA) in 2% PFA, according to the manufacturer’s instructions. Differentiated cells were incubated in staining mixture (1× cell viability indicator and 1× cell membrane stain) with 2% PFA for 20 min at 37 °C. Subsequently, the staining solution was removed, and the culture plates were filled with 1× background suppression dye in 1× PBS. Neurite outgrowth (λex555 nm/λem565 nm) and cell viability (λex495 nm/λem515 nm) were visualized with an Eclipse Ts2 fluorescent microscope (Nikon, Tokyo, Japan). Fluorescence intensities were analyzed using ImageJ software.

### 2.11. Neurite Complexity Analyses and Sholl Analyses

SH-SY5Y cells (4 × 10^4^ cells/cm^2^) and PCNs (1.4 × 10^4^ cells/cm^2^) were seeded to measure neurite complexity (neurite length, number of secondary branches, and neurites from soma of single neurons), using ImageJ software. All measured data were normalized to control group data.

For morphological analyses of green fluorescent protein (GFP)-transfected PCNs, neuronal branches were measured on neuron images. Neurite branching was measured using the Sholl analysis plugin of ImageJ. We located the soma of the selected neuron on the start radius, and measured dendrite intersections from 10 to 1070 μm per 10 μm step size. All samples were sets of segmentation with 3 per radius, and a polynomial fit for “best fitting degree”.

### 2.12. Dendritic Spine Analyses

For the dendritic spine analysis, morphological analyses of dendritic spine classifications were performed on more than 20 neurites per GFP-transfected neurons. The standard spine shapes were defined as follows: Filopodia (length >2 μm), Long thin (length <2 μm with spine head <0.6 μm in width), Thin (length <1 μm with spine head <0.6 μm in width), Stubby (length:width ratio <1), Mushroom (spine head >0.6 μm in width), and Branched (≥2 spine heads). Imaged dendritic spines were classified according to spine shape per 10 μm, using ImageJ.

### 2.13. Statistical Analyses

Statistical analyses were performed using Prism 8 (GraphPad Software Inc., San Diego, CA, USA). The data were expressed as the group mean ± standard error of mean. Ordinary one-way ANOVA with Bonferroni correction and unpaired two-tailed *t*-tests with Welch’s correction were used. *P*-value was decided as n.s > 0.05, * *p* < 0.05, ** *p* < 0.01, and *** *p* < 0.001.

## 3. Results

### 3.1. Ex-4 Suppressed Apoptosis in Neurons under PA-Induced Oxidative Stress

**Figure 1:**
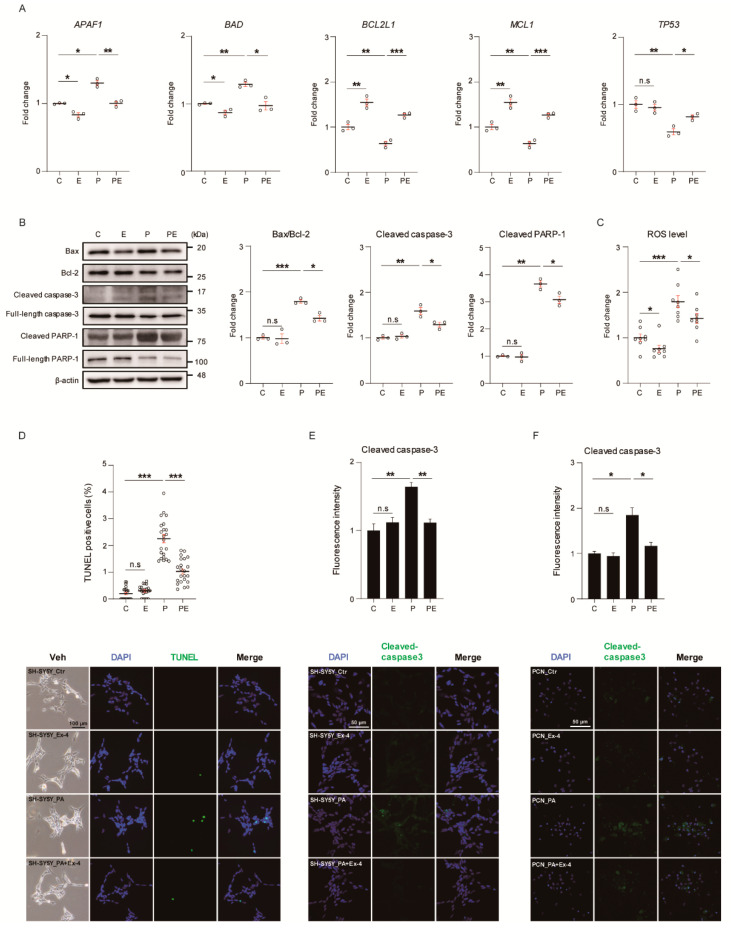
Exendin-4 suppresses the neuronal apoptosis under palmitic acid-induced oxidative stress. (**A**) Comparison of mRNA expression levels of pro-apoptotic (*APAF1* and *BAD*) and anti-apoptotic genes (*BCL2L1*, *MCL1*, and *TP53*) in SH-SY5Y cells treated with Vehicle (C or Ctr), Exendin-4 (E or Ex-4), Palmitic acid (P or PA), and both Palmitic acid and Exendin-4 (PE or PA+Ex-4). The mRNA level of each gene is normalized to *GAPDH* level. (**B**) Western blot for the protein level of neuronal apoptosis signaling in SH-SY5Y cells treated with reagents described in (**A**). Each protein level is normalized to β-actin. The cleaved proteins were normalized to each full-length protein. (**C**) Measurement of ROS level stained with DCF-DA (100 μM) in SH-SY5Y cells treated with reagents described in (**A**). (**D**) Detection of PA-induced apoptosis by transferase dUTP nick end labeling (TUNEL) assay in SH-SY5Y cells treated with reagents described in A. Nuclei were counterstained with DAPI (blue), and apoptotic cell stained with TUNEL (green). The percentage of TUNEL positive cells of each group is normalized to the vehicle (control) group; >100 cells per group were analyzed. Scale bar: 100 μm. (**E**,**F**) immunocytochemistry images of cleaved caspase-3 expression in SH-SY5Y and primary cortical neuron (PCN) neurons at day 7 in vitro (DIV 7) treated with reagents described in A. Nuclei were counterstained with DAPI (blue), and apoptotic cell stained with cleaved caspase-3 (green), >100 (**E**) and >50 (**F**) cells per group were analyzed. Scale bar: 50 μm. Data information: SH-SY5Y cells were treated with 10 nM of Exendin-4 and 50 μM of palmitic acid per group, according to the treatment plan (Figure S1B,C). In (**A**–**F**), error bars represent S.E.M. from three independent experiments. n.s *p* > 0.05, * *p* < 0.05, ** *p* < 0.01, *** *p* < 0.001 (unpaired two-tail *t*-tests with Welch’s correction).

We investigated whether apoptosis response was suppressed by Ex-4 in PA-treated neurons (Figure 1). The treatment concentrations were decided based on our previous test (Figure S2), conducted according to the treatment plan (Figure S1A). The PA concentration was selected as 50 μM, which significantly increased the expression change of the apoptosis-related proteins (Figure S2A). The concentration of Ex-4 was determined as 10 nM, which reduced the expression change of PA-induced apoptosis-related proteins, and significantly improved neuronal complexity and neuronal differentiation marker gene expression (Figure S2B–D). We measured the mRNA levels of pro-apoptosis-related and anti-apoptosis-related genes in SH-SY5Y cells under PA-induced oxidative stress. Our data showed that the expression of pro-apoptotic genes, such as apoptotic peptidase activating factor 1 (*APAF1*) and BCL2-associated agonist of cell death (*BAD*) were increased, whereas those of anti-apoptotic genes such as BCL2 like 1 (*BCL2L1*), myeloid cell leukemia sequence 1 (*MCL1*), and tumor protein p53 (*TP53*) were decreased (Figure 1A). Ex-4 treatment inhibited the expression of pro-apoptotic genes including *APAF1* and *BAD*, and increased the expression of anti-apoptotic genes, such as *BCL2L1*, *MCL1*, and *TP53* in PA-treated neurons (Figure 1A).

To investigate the regulatory role of Ex-4 in the apoptosis signaling, we examined the levels of apoptosis-related proteins in Ex-4-exposed neurons under PA-induced apoptosis (Figure 1B). The expression of apoptosis signaling-associated proteins such as Bax/Bcl-2, cleaved caspase-3 and cleaved PARP-1 was increased in neurons under PA-induced oxidative stress. Ex-4 inhibited the expression of apoptosis-related proteins in PA-treated neurons, while Ex-4-only treatment did not alter the basal level of these proteins in control neurons.

Additionally, ROS production was elevated in neurons under PA-induced oxidative stress compared to control (vehicle-treated) neurons (Figure 1C). Ex-4 attenuated the increased ROS levels in neurons despite the PA-induced oxidative stress, and suppressed ROS levels in the control group. To investigate the effect of Ex-4 on neurons in apoptotic cell death response, we performed TUNEL staining (Figure 1D). TUNEL-positive cells increased in number in PA-treated cultures compared with control cultures. However, TUNEL-positive cells decreased in number with Ex-4 treatment in PA-induced oxidative stress conditions. Ex-4 did not affect the number of TUNEL-positive cells in neurons under normal conditions.

To confirm the expression of apoptotic markers in neurons, we observed the expression of cleaved-caspase3 by immunocytochemistry (Figure 1E,F). Compared to control cells, the fluorescence intensity increased both in SH-SY5Y cells and PCNs under PA-induced oxidative stress conditions. These data indicate that the apoptotic cell death of SH-SY5Y cells and PCNs under PA-induced oxidative stress was inhibited by Ex-4 treatment.

### 3.2. Ex-4 Improved Insulin Signaling in Neurons under PA-Induced Oxidative Stress

**Figure 2:**
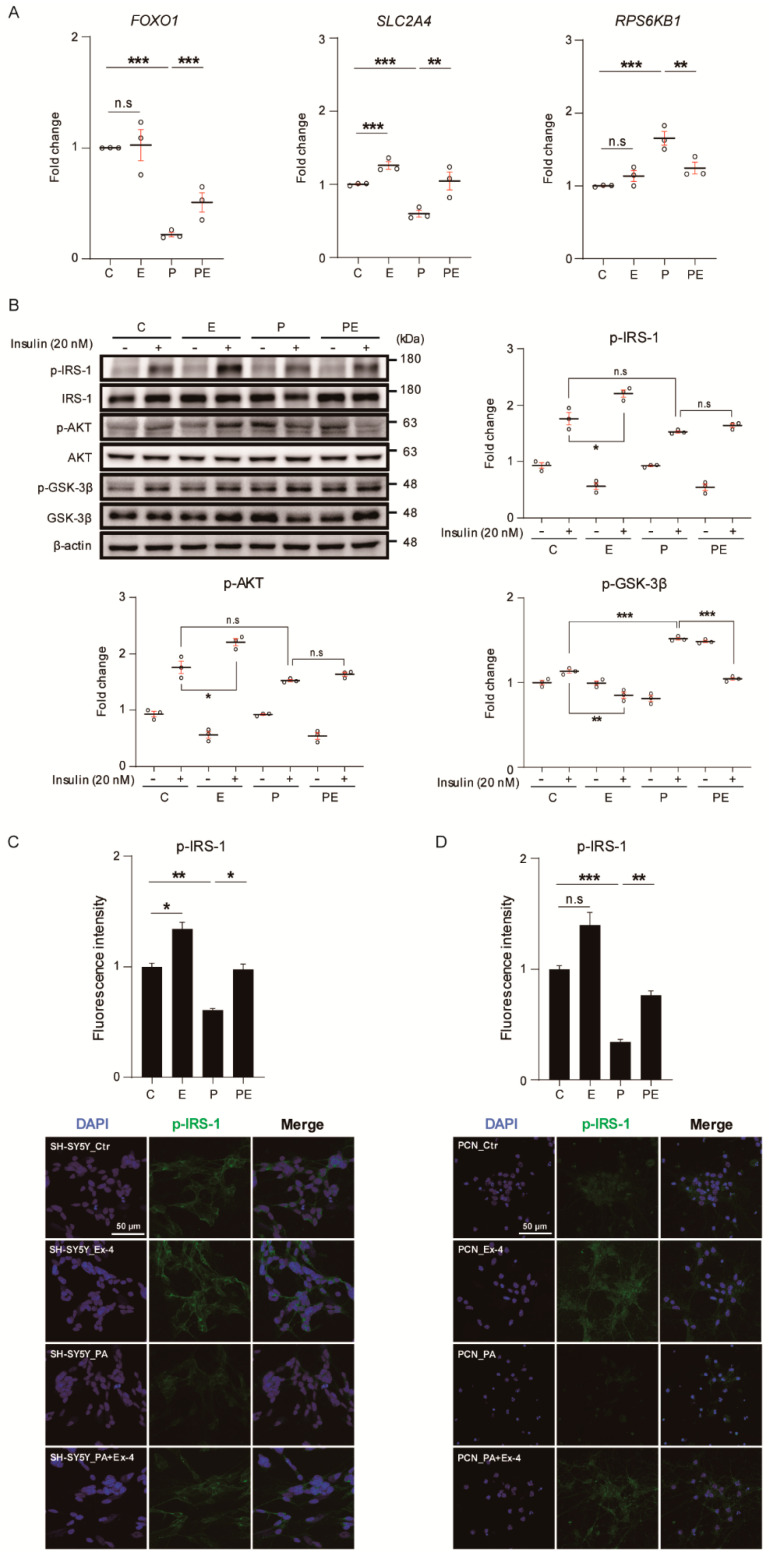
Exendin-4 improves insulin signaling in a neuron under palmitic acid-induced oxidative stress. (**A**) Comparison of mRNA expression levels of insulin signaling related genes (*FOXO1*, *SLC2A4*, and *RPS6KB1*) in SH-SY5Y cells treated with vehicle (C or Ctr), Exendin-4 (E or Ex-4), palmitic acid (P or PA), and both palmitic acid and Exendin-4 (PE or PA+Ex-4). The mRNA level of each gene is normalized to *GAPDH* level. (**B**) Western blot of insulin signaling through 20 nM insulin stimulation for 10 min in SH-SY5Y cells treated with reagents described in A. Each protein level is normalized to β-actin. The pIRS-1, pAKT, and pGSK-3β levels were normalized to IRS-1, AKT, and GSK-3β of total form. (**C**,**D**) immunocytochemistry images of pIRS-1 expression in SH-SY5Y cells and PCN neurons at DIV 7 treated with reagents described in A. Nuclei were counterstained with DAPI (blue) and insulin receptor stained with pIRS-1 (green), >100 (**E**) and >50 (**F**) cells per group were analyzed. Scale bar: 50 μm. Data information: SH-SY5Y cells were treated with 10 nM of Exendin-4 and 50 μM of palmitic acid per group according to the treatment plan (Figure S1B,C). In (**A**–**D**), error bars represent S.E.M. from three independent experiments. n.s *p* > 0.05, * *p* < 0.05, ** *p* < 0.01, *** *p* < 0.001 (unpaired two-tail *t*-tests with Welch’s correction).

Insulin binds to insulin receptors such as IRS1, and mainly regulates cell metabolism and glucose transport through AKT signaling [25]. We investigated whether Ex-4 inhibits insulin resistance caused by elevated PA concentration in neurons through the AKT signaling, using the treatment plan shown in Figure S1B. The mRNA expression of insulin signaling-associated genes such as Forkhead Box O1 (*FOXO1*) and trafficking regulator of GLUT4 (*SLC2A4*) was significantly decreased in neurons by PA treatment (Figure 2A). The expression level of the ribosomal protein S6 kinase B1 (*RPS6KB1*) was increased in neurons under PA-induced oxidative stress compared with control cells. However, Ex-4 reversed alteration in the mRNA expression of these genes in neurons caused by PA-induced insulin resistance. The basal levels of *FOXO1* and *RPS6KB1* were not altered by Ex-4 compared to controls, though *SLC2A4* expression was increased by Ex-4 treatment.

To investigate whether Ex-4 regulates insulin signaling in neurons under PA-induced oxidative stress, we treated neurons with 20 nM insulin for 15 min and analyzed them by western blotting (Figure 2B). The phosphorylation of IRS-1 was not different among the groups. The phosphorylation of AKT was downregulated, and that of GSK-3β was upregulated in neurons under PA-induced insulin resistance. However, this reduced insulin sensitivity was recovered by Ex-4 treatment. In addition, Ex-4 improved the basal expression of IRS-1 and GSK-3β phosphorylation in neurons under PA-induced insulin resistance, compared to the control group. Reduction of FOXO1 mRNA expression under insulin resistance states leads to the reduction of AKT phosphorylation, followed by abnormal activation of GSK, which regulates glycogen synthesis [26]. However, Ex-4 suppressed PA-induced insulin resistance by enhancing the insulin signaling related to insulin response.

We confirmed the expression of insulin receptors in SH-SY5Y cells and PCNs (Figure 2C,D, respectively). Both cell types showed significantly reduced phosphorylation of IRS-1 under PA-induced insulin resistance conditions. However, Ex-4 treatment increased the expression of phosphorylated IRS-1 in neurons under PA-induced insulin resistance. These data indicate that Ex-4 inhibited insulin resistance, and regulated insulin signaling in neurons under PA-induced insulin resistance conditions.

### 3.3. Ex-4 Improved Mitochondrial Function in Neurons against PA-Induced Oxidative Stress

**Figure 3:**
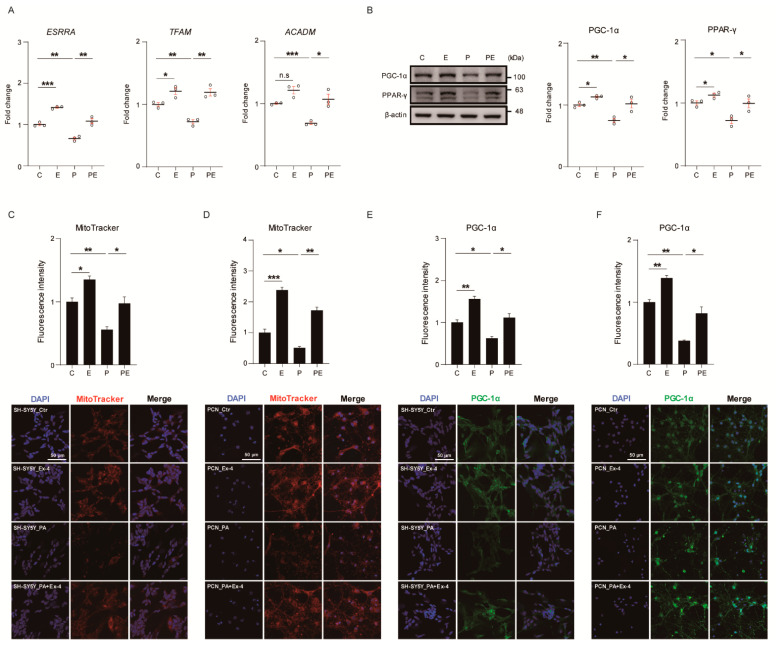
Exendin-4 improves the mitochondrial function of neurons against palmitic acid-induced oxidative stress. (**A**) Comparison mRNA expression of mitochondrial biogenesis *ESRRA*, mitochondrial transcription factor *TFAM*, and *ACADM* enzyme in SH-SY5Y cells treated with vehicle (C or Ctr), Exendin-4 (E or Ex-4), palmitic acid (P or PA), and both palmitic acid and Exendin-4 (PE or PA+Ex-4). The mRNA level of each gene is normalized to *GAPDH* level. (**B**) Western blot of metabolic process related protein expression in SH-SY5Y cells treated with reagents described in A. Each protein level is normalized to β-actin. (**C**,**D**) Fluorescent images of mitochondrial membrane potential with specific dye Mito-tracker (200 nM) in SH-SY5Y cells, and PCN neurons at DIV7 treated with reagents described in A. Nuclei were counterstained with DAPI (blue) and active mitochondria stained with MitoTracker deep red (red), >100 (E) and >50 (F) cells per group were analyzed. Scale bar: 50 μm. (**E**,**F**) immunocytochemistry images of PGC-1α expression in SH-SY5Y cells, and PCN neurons at DIV7 treated with reagents described in A. Nuclei were counterstained with DAPI (blue) and insulin receptor stained with PGC-1α (green), >100 (**E**) and >50 (**F**) cells per group were analyzed. Scale bar: 50 μm. Data information: SH-SY5Y cells were treated with 10 nM of Exendin-4 and 50 μM of palmitic acid per group, according to the treatment plan (Figure S1B,C). In (**A**–**F**), error bars represent S.E.M. from three independent experiments. n.s *p* > 0.05, * *p* < 0.05, ** *p* < 0.01, *** *p* < 0.001 (unpaired two-tail *t*-tests with Welch’s correction).

PA undergoes fatty acid oxidation in the mitochondria leading to adenosine triphosphate (ATP) production [27]. However, excessive PA accumulation in neurons causes ER stress and mitochondrial dysfunction, such as mitochondrial ROS production [27]. To confirm whether Ex-4 ameliorates the mitochondrial dysfunction caused by PA-induced oxidative stress, we measured the mRNA expression of genes related with mitochondrial biogenesis, transcription factors, and energy production-related enzymes (treatment plan, Figure S1B) (results, Figure 3A). The mRNA expression of genes such as estrogen-related receptor alpha (*ESRRA*), transcription factor A, mitochondrial (*TFAM*), and acyl-coenzyme A dehydrogenase (*ACADM*) was significantly downregulated in PA-treated neurons, compared to control cells. However, Ex-4 increased the mRNA expression of these genes in PA-treated neurons. The basal expression of *ESRRA* and *TFAM* was increased by Ex-4 compared with controls, whereas there was no change in the mRNA level of the *ACADM* gene in PA-treated neurons. To identify whether or not Ex-4 can improve mitochondrial biogenesis and metabolism, we measured the expression changes of related proteins by western blot analysis (Figure 3B). PA downregulated the expression of PGC-1α and PPAR-γ, which are involved in mitochondrial dysfunction in neurons. Ex-4 improved the expression of these proteins, compared with control neurons. The basal levels of PGC-1α and PPAR-γ were increased in normal conditions. Additionally, we measured the mitochondrial density in PA-treated neurons using the MitoTracker live cell assay (Figure 3C,D). The fluorescence intensity data showed a reduction of mitochondrial dysfunction in Ex-4 treatment both in SH-SY5Y cells and PCNs, compared to controls. Moreover, the fluorescence intensity of Ex-4-treated cells was higher in comparison with control cells. We measured PGC-1α protein levels in both cell types (Figure 3E,F) by western blotting. Mitochondrial biogenesis-related protein expression was increased in Ex-4-treated cells despite the PA-induced oxidative stress, as observed by western blotting, as well as by immunostaining. Ex-4-treated neurons showed increased PGC-1α (fluorescence intensity data) compared to controls. Taken together, we may conclude that Ex-4 enhances mitochondrial function in neurons under PA-induced oxidative stress, as well as in neurons under normal conditions.

### 3.4. Ex-4 Improved the Structure and Differentiation in Neurons under PA-Induced Oxidative Stress

**Figure 4:**
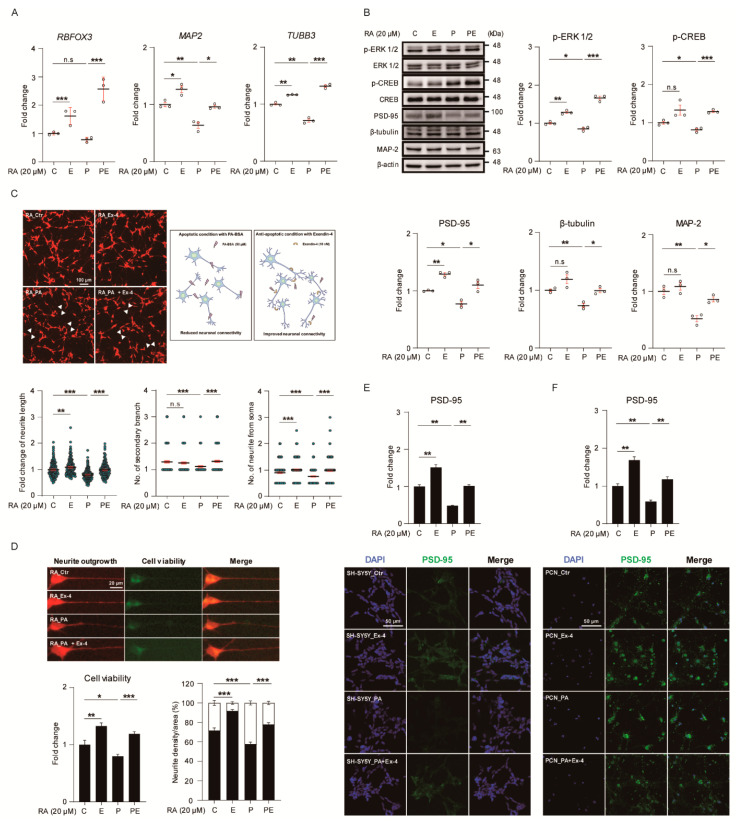
Exendin-4 improves the neural structure by promoting neural differentiation under palmitic acid-induced oxidative stress. (**A**) Comparison mRNA expression of gene-related with neural differentiation (*RBFOX3*, *TUBB3*, and *MAP2*) in SH-SY5Y cells treated with vehicle (C or Ctr), Exendin-4 (E or Ex-4), palmitic acid (P or PA), and both palmitic acid and Exendin-4 (PE or PA+Ex-4). The mRNA level of each gene is normalized to *GAPDH* level. (**B**) Western blot of neural differentiation signaling was promoted by Ex-4 treatment in SH-SY5Y cells treated with reagents described in A. Each protein level is normalized to β-actin. The phosphorylation of pERK 1/2 and pCREB is normalized to ERK 1/2 and CREB total form, respectively. (**C**) Fluorescent images of RA-induced differentiated SH-SY5Y cells treated with reagents described in A. Scale bar: 100 μm. Neurite complexity (neurite length, number of secondary branches, and neurite from soma) was analyzed from >150 cells per group. The white point indicates PA-induced damaged neurite site and recovered neurite of Ex-4 treated group. (**D**) Fluorescent images of cell viability (green) and neurite shrinkage (red) in RA-induced differentiated SH-SY5Y cells treated with reagents described in A. Scale bar: 10 μm. (**E**,**F**) immunocytochemistry images of PSD-95 expression in SH-SY5Y cells and PCN neurons on day 21 in vitro (DIV21) treated with reagents described in A. Nuclei were counterstained with DAPI (blue) and insulin receptor stained with PSD-95 (green), >100 (**E**) and >50 (**F**) cells per group were analyzed. Scale bar: 50 μm. Data information: SH-SY5Y cells were treated with 10 nM of Exendin-4 and 50 μM of palmitic acid per group according to the treatment plan (Figure S1B,D) In (**A**–**F**), error bars represent S.E.M. from three independent experiments. n.s *p* > 0.05, * *p* < 0.05, ** *p* < 0.01, *** *p* < 0.001 (unpaired two-tail *t*-tests with Welch’s correction).

We examined whether or not Ex-4 increases gene expression related to neurogenesis-, axon guidance-, and microtubule-stabilization-associated genes using the treatment plan shown in Figure S1B. The expression of RNA binding fox-1 homolog 3 (*RBFOX*), tubulin beta 3 III (*TUBB3*), and *MAP2* was upregulated in neurons under normal conditions by Ex-4 treatment (Figure 4A). However, the expression of *TUBB3* and *MAP2* was downregulated in PA-treated neurons. Ex-4 treatment significantly inhibited PA-induced neuronal damage, and increased the expression of matured neuronal markers such as *RBFOX3*, *MAP2*, and *TUBB3*. We investigated the expression of neurite growth-related proteins in PA-treated neurons (Figure 4B). The phosphorylation and expression of these proteins were decreased with PA treatment, compared to controls. However, Ex-4 enhanced the reduced phosphorylation and protein level of these genes in neurons caused by PA treatment. The basal levels of ERK1/2 phosphorylation and PSD-95 expression were increased in neurons by Ex-4 treatment compared to controls. Taken together, Ex-4 regulates the expression of mRNA and protein related to neurite outgrowth through ERK/CREB signaling. Furthermore, we investigated whether Ex-4 improves neuronal structure in neurons under PA-induced oxidative stress conditions, using SH-SY5Y differentiated with RA. Compared to controls, the neurite complexity of PA-treated neurons was significantly reduced, based on neurite length and the number of neurites and secondary branches (Figure 4C). However, compared to the treatment using PA alone, PA+Ex-4 treatment resulted in longer neurite length. Furthermore, Ex-4 enhanced neurite outgrowth and the number of neurite branches compared to neurons under normal conditions. In undifferentiated cells without RA treatment, PA reduced neurite length and the number of secondary branches, compared to controls (Figure S3C). However, Ex-4 improved all neurite parameters and neurite outgrowth parameters in PA-treated neurons (Figure S3B), compared to controls (Figure S3A). Neuronal connectivity was weakened in PA-treated neurons (white pointed areas (Figure S3A,B). However, neuronal connections were rescued by Ex-4 treatment in spite of PA-induced oxidative stress. We measured the structure and process changes in PA-treated neurons (Figure 4D). Cell viability and PA-reduced neurite shrinkage were reduced in PA-treated neurons compared to controls. However, Ex-4 restored viability and shrinkage of neurons under PA-induced oxidative stress.

We evaluated the expression of PSD-95 in SH-SY5Y cells and PCNs under PA-induced oxidative stress (Figure 4E,F), and observed lower PSD-95 expression (immunofluorescence) compared to control cells. Ex-4 reversed the reduction in fluorescence intensity in PA-treated neurons. Ex-4 enhanced the basal level of PSD-95 intensity in neurons under normal conditions. These data show that Ex-4 ameliorated neuronal complexity and regulated neurite-related protein expression in PA-treated neurons.

### 3.5. Ex-4 Improved Synaptic Plasticity in Neurons under PA-Induced Oxidative Stress

**Figure 5:**
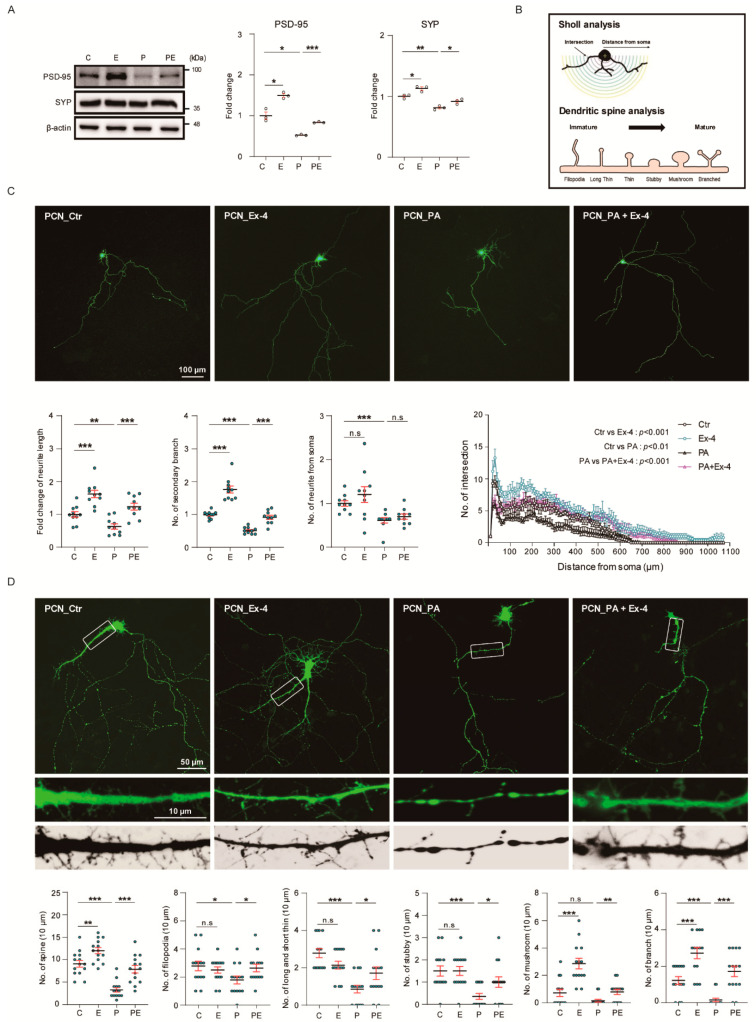
Exendin-4 improved the synaptic plasticity under palmitic acid-induced oxidative stress. (**A**) Western blot of synaptic plasticity-related protein in PCN at DIV21 treated with vehicle (C or Ctr), Exendin-4 (E or Ex-4), palmitic acid (P or PA), and both palmitic acid and Exendin-4 (PE or PA+Ex-4). Each protein level is normalized to β-actin. (**B**) Scheme of Sholl analysis (upper) and dendrite spine morphology analysis (lower). (**C**) Fluorescent images, histograms, and Sholl analysis for neurite complexity of pMax-GFP-transfected PCN neurons at DIV7 treated with reagents described in (**A**). Scale bar: 100 μm. Neurite length, number of neurites from soma, and Sholl analysis analyzed from >150 cells per group. (**D**) Fluorescent images for dendritic spine morphology of pMax-GFP-transfected PCN neurons at DIV21 treated with reagents described in A. Scale bars: 50 μm (GFP transfected neuron) and 10 μm (dendrite spine morphology). Dendritic spine morphology, such as filopodia, long thin and short thin, stubby, mushroom and branched morphology were analyzed from >100 cells per group. Data information: SH-SY5Y cells were treated with 10 nM of Exendin-4 and 50 μM of palmitic acid per group according to the treatment plan (Figure S1C,D). In (**A**–**D**), error bars represent S.E.M. from three independent experiments. n.s *p* > 0.05, * *p* < 0.05, ** *p* < 0.01, *** *p* < 0.001 (unpaired two-tail *t*-tests with Welch’s correction).

We examined the effect of Ex-4 on synaptic plasticity in PCNs. The expression of PSD-95 and synaptophysin proteins related to synapse formation was decreased in neurons by PA treatment (Figure 5A) (treatment plan, Figure S1C,D). However, Ex-4 restored the PSD-95 and synaptophysin protein levels in PA-treated neurons, and increased the basal levels of these genes in neurons in normal conditions. Figure 5B shows a brief scheme of neurite complexity and dendritic spine morphology analysis (Figure 5B). Neurite lengths, numbers of neurites from soma, and secondary branches significantly decreased in PA-treated neurons (Figure 5C). Ex-4 treatment resulted in an increase in neurite length and the number of secondary branches in PA-treated neurons, as well as in control neurons. Neuritic complexity (as per Sholl analysis) was considerably decreased in PA-treated PCNs compared to control PCNs. We found that Ex-4 enhanced the neural complexity in PA-treated neurons, as well as in control neurons. We confirmed that Ex-4 contributes to a morphological change in the dendritic spines involved in neurite complexity in PCNs (Figure 5D). PA-treated neurons significantly decreased the number of dendritic spines, and showed long and short thin, stubby, and branch shaped spines compared to control neurons. However, PA+Ex-4 treatment group neurons showed an increased number of dendritic spines, filopodia, long and short thin, stubby, and branch shaped spines compared to PA-treated group. In addition, Ex-4 increased the number of dendritic spines, and changed spine forms to mushroom, and branch shaped spines compared to control group. Ex-4 could boost the new formation of spines and spine maturation in neurons under PA-induced neuronal damages. Therefore, our results showed Ex-4 accelerates the synaptic plasticity, neurite complexity, and spine maturation both under normal and neuronal damaged conditions.

## 4. Discussion

In this study, we investigated how Ex-4, a GLP-1 agonist, contributes to neuronal function in PA-induced obesity-mimicking conditions. Four significant findings from the present study are given below.

First, our results showed that Ex-4 attenuated neuronal cell death in in-vitro PA-induced oxidative stress conditions, given that PA induces oxidative stress (ROS generation) and promotes cell death signaling in neurons [28]. Ex-4 reduced the mRNA levels of pro-apoptotic factors, such as *APAF1* [29] and *BAD* [30], and induced the mRNA levels of anti-apoptotic genes, including *BCL2* [31] and *MCL1* [31], under PA-induced oxidative stress.

The elevation of BCL-2 protein and *MCL1* mRNA level by Ex-4 treatment under PA-induced oxidative stress condition shows that Ex-4 inhibits the intrinsic mitochondrial cell death pathway in SH-SY5Y cells and PCNs. Additionally, our data showed a decrease in the protein levels of the pro-apoptotic factor, BAX, cleaved caspase-3, and cleaved PARP-1, signifying a reduction of apoptotic signaling by Ex-4 in neurons. The reduction of cleaved PARP-1 implies decreased DNA chain breakdown and damage [32] in Ex-4-treated neurons. Reduced TUNEL-positive cells also imply the inhibition of DNA cleavage in neurons [33] by Ex-4; decreased cleaved caspase-3 levels also signify decreased caspase-3-dependent apoptosis in neurons [34] treated with Ex-4. ROS derived mitochondria [35] are involved in cellular metabolism [36], regulation of neuronal polarization, neuronal differentiation, neuronal apoptotic signal activation, neurite outgrowth, and synaptic plasticity [37,38]. Consequently, the reduction of ROS in PA-treated neurons by Ex-4 may indicate the inhibition of pro-apoptotic signaling, initiation of neurite outgrowth, and repair of synaptic plasticity.

Second, we found that Ex-4 improved insulin sensitivity in PA-treated neurons. Insulin binds to the insulin receptor and promotes the phosphorylation of IRS in the brain [39]. The degradation of IRS in neurons induces insulin resistance in the brain [40]. Recent studies have demonstrated that abnormal serine phosphorylation of IRS-1 is commonly observed in the brain of AD patients [41], and the reduced expression of IRS-1 results in insulin resistance in the brain [42]. Based on these results, Ex-4 may promote increased IRS-1 expression and improve insulin resistance in the obese brain. Our data showed that Ex-4 could increase the mRNA level of *FOXO1* and *SLC2A4* in neurons, in spite of PA-induced oxidative stress. *FOXO1* is commonly expressed in brain hippocampal formations (such as the dentate gyrus and ventral hippocampus, and the striatum), especially in areas related to memory function [43]. It has been reported to regulate insulin action in neurons [44], and may be involved in obesity-induced memory loss [45]. Additionally, *RPS6KB1* has been known to control glucose metabolism and insulin resistance in neurons [46]. Furthermore, insulin could inhibit tau phosphorylation in neurons by controlling the activity of GSK-3β through AKT signaling [47]. Insulin resistance results in the abnormal activation of GSK-3β, and subsequently an increase of tau phosphorylation, leading to neuronal dysfunction in the brain [48]. Based on these findings, it can be concluded that Ex-4 promotes insulin sensitivity in neurons under obese conditions by attenuating the activation of GSK-3β and AKT signaling, suggesting that PA-induced oxidative stress condition leads to AKT/NF-κB signaling activation [17]. Thus, Ex-4 improves insulin sensitivity in neurons under obesity-induced hyperlipidemia conditions. Although we did not perform memory-related behavioral tests, we expect that Ex-4 will improve cognitive function by promoting insulin sensitivity in the obese brain.

Third, we found that Ex-4 ameliorates mitochondrial function in neurons under PA-induced oxidative stress. While mitochondria are the main sources of ATP in neurons, they are also sources of ROS in stress conditions, and contribute to neuropathological signaling [49,50].

Mitochondrial biogenesis involves PGC-1α [51] and the activation of the expression of *TFAM* as a co-factor [52]. PGC-1α is linked to the activation of the CREB and FOXO transcription factors in the mitochondria [53]. It upregulates the expression of transcription factors such as the *ESRRA* [54], several antioxidant enzymes [55], and mammalian homolog of tribble 3 (TRB-3) nuclear genes [56]. Our results showed that Ex-4 treatment increased PGC-1α activity, mitochondrial density [57], and the activation of transcription factors, such as *ESRRA* and *TFAM*, in neurons under PA-induced oxidative stress.

We also observed that Ex-4 boosts expression of PPAR-γ in neurons under PA-induced oxidative stress. PPAR-γ is expressed in several brain regions such as the hippocampus dentate gyrus and cortex [58], and its activation exerts neuroprotective effects against neurotoxicity [59]. Moreover, the activation of PPAR-γ accelerates mitochondrial biogenesis and inhibits neuronal loss against damage condition [58]. Accordingly, we assume that Ex-4 improves mitochondrial function by promoting mitochondrial biogenesis and mitochondrial density in the obese brain.

Fourth, we observed that Ex-4 ameliorated neuronal connectivity and synaptic plasticity in PA-induced oxidative stress. Increased *RBFOX3*, *TUBB3*, and *MAP2* expression with Ex-4 treatment, in spite of PA-induced oxidative stress, was found in the study. *RBFOX3* has been reported to promote neurogenesis and strengthen synaptic plasticity [60]. *TUBB3* (neuronal tubulin protein) is involved in axon growth cones, increases neurite length, and contributes to the recovery of neuronal function and connectivity [61]. MAP-2 is a major neuronal cytoskeleton protein promoting microtubule stabilization during neuronal differentiation and development [62]. These findings suggest that Ex-4 promotes stable neuronal connectivity and boosts neuronal differentiation in the obese brain.

Our findings imply that Ex-4 enhances synaptic plasticity by regulating synaptic density proteins in the obese brain, suggesting that synaptophysin and PSD-95 proteins are related to neuronal differentiation, synapse maturation, and neuronal function [63].

In our study, we found that Ex-4 activated ERK and CREB phosphorylation in neurons under PA-induced oxidative stress.

The activation of ERK phosphorylation results in the improvement of long-term potentiation and the enhancement of memory function [64], while the activation of ERK and CREB phosphorylation promotes neurite growth [65]. It has been reported that liraglutide (GLP-1 receptor agonist) induces the activation of ERK phosphorylation [66]. Considering these points, Ex-4 boosts neurite growth and ameliorates memory function in the obese brain. We also found that Ex-4 boosts dendritic spine maturation, as well as neurite elongation, in neurons under PA-induced oxidative stress. Dendritic spine maturation implies neuronal maturation and stable synaptic plasticity [67]. The dendrite morphology and type of neurite processes reflect the stability of neuronal connectivity and neurite elongation and axon outgrowth [68]. Additionally, proper neurite branching and appropriate reorganization of axonal microtubules results in the formation of stable neuronal circuits [69]. Poor neurite branching morphogenesis and impairment of axonal elongation, related to cytoskeletal reorganization [70], leads to neurodegenerative and neuropsychiatric diseases, accompanied by cognitive decline and impaired sensory perception [71]. Several proteins such as actin [72] and FOXO [73] contribute directly to neuronal morphogenesis, dendritic branching, and stable neuronal connectivity remodeling [74]. In addition, dendritic spine morphology contributes to the memory function by affecting synaptic plasticity [75,76]. Several studies have mentioned that the morphology and number of dendritic spines influences memory processes and behavior patterns [77,78]. Our data showed that Ex-4 strengthens the spine head of PCNs, promotes a mushroom-shaped spine morphology (considered as the mature form of the spine [79]), and induces an increase in spine numbers in neurons under PA-induced oxidative stress.

## 5. Conclusions

In conclusion, we elucidated the role of Ex-4 in neurons under PA-induced obesity conditions. Although previous studies reported that Ex-4 has neuroprotective effects and promotes neurite growth [80,81], its role in the obese brain remains unclear.

Thus, we propose that Ex-4 may be a therapeutic solution to cure neuropathology in patients with obesity.

## Data Availability

The data presented in this study are available within the article. Other data related to this study are available on request from the corresponding author.

## Supplementary Materials

The following are available online at https://www.mdpi.com/2076-3921/10/1/78/s1, Figure S1: Experimental schemes and primer lists of qRT-PCR. Figure S2: Identification of palmitic acid and exendin-4 treat concentration in SH-SY5Y cells. Figure S3: Comparison of fluorescent images and histograms of undifferentiated- and differentiated SH-SY5Y cells.

## Abbreviations

GLP-1	glucagon like peptide 1
PA	palmitic acid
AD	Alzheimer’s disease
ER	endoplasmic reticulum
PCNs	primary cortical neurons
Ex-4	exendin-4
*APAF1*	apoptotic peptidase activating factor 1
*BAD*	BCL2-associated agonist of cell death
*BCL2L1*	BCL2 like 1
*MCL1*	myeloid cell leukemia sequence 1
*TP53*	tumor protein p53
*FOXO1*	Forkhead Box O1
*RPS6KB1*	ribosomal protein S6 kinase B1
IRS-1	Insulin receptor substrate 1
Bax	BCL2-associated X apoptosis regulator
Bcl-2	B-cell lymphoma 2
Caspase-3	cysteine-aspartic acid protease 3
AKT	phosphatidylinositol kinase (PI3K)/protein kinase B
GSK-3β	glycogen synthase kinase 3 beta
PPAR-γ	peroxisome proliferator-activated receptor gamma
ERK1/2	p44/42 MAPK extracellular signal-regulated kinases 1/2
CREB	cAMP-response element binding protein
PSD-95	postsynaptic density protein 95
PARP-1	Cleaved-Poly [ADP-ribose] polymerase 1
PGC-1α	peroxisome proliferator-activated receptor gamma coactivator 1-alpha
MAP-2	microtubule-associated protein 2, ROS, reactive oxygen species
GFP	green fluorescent protein
ATP	adenosine triphosphate
*ESRRA*	estrogen-related receptor alpha
*TFAM*	transcription factor A, mitochondrial
*ACADM*	acyl-coenzyme A dehydrogenase
*RBFOX*	RNA binding fox-1 homolog 3
*TUBB3*	tubulin beta 3 III
