# Supplementary material for: Role of Exendin-4 in Brain Insulin Resistance, Mitochondrial Function, and Neurite Outgrowth in Neurons under Palmitic Acid-Induced Oxidative Stress
Source: Antioxidants (Basel). 2021 Jan 9;10(1):78. doi: 10.3390/antiox10010078 (PMC7827489; doi:10.3390/antiox10010078)
Supplement: Supplementary file 1 [file antioxidants-10-00078-s001.pdf]

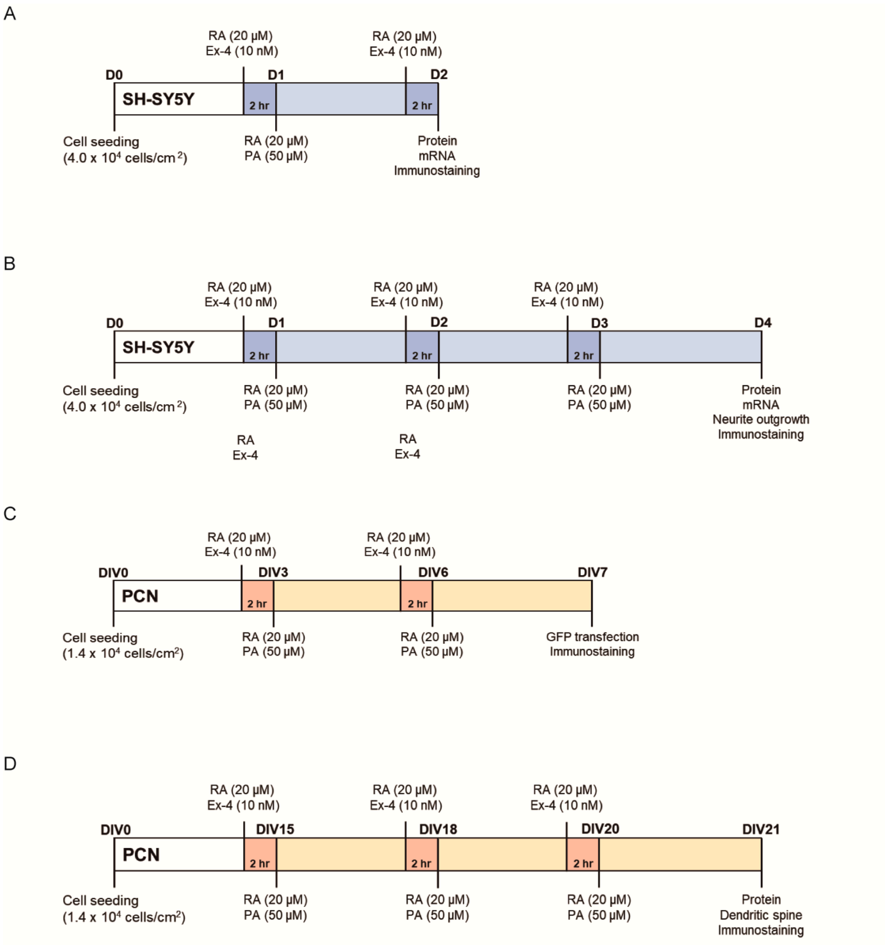

E

	Forward	Reverse
APAF1	5'-AAACTCCTGTTGCCGTTTCC-3'	5'-CTGAGCTGTCAACCATGAGC-3'
BAD	5'-CTCCACATCCCGAAACTCCA-3'	5'-GAGACAGCACGGATCCTCTT-3'
BCL2L1	5'-GGACAATGGACTGGTTGAGC-3'	5'-GGCCTCAGTCCTGTTCTCTT-3'
MCL1	5'-GGGAAGCTATGGAGGAGGAC-3'	5'-ACACAGGTCACTGGCATTCT-3'
TP53	5'-CTGGCATTTGCACCTACCTC-3'	5'-TAACCAGCTGCCCAACTGTA-3'
FOXO1	5'-CGCTACCCGAGTTTAGTAACAG-3'	5'-TCAACATCAGGCACTTCTCAG-3'
SLC2A4	5'-GGCAAGAGGGAACAGGAAAG-3'	5'-CAGGAAGGGAGAGGAAGAAAG-3'
RPS6KB1	5'-ACAGCAAATCCTCAGACACC-3'	5'-TGCTTCCCCACTCATTGTC-3'
ESRRA	5'-GGGCATTGAGCCTCTCTACA-3'	5'-CTGTCTCCGAGGAACCCTTT-3'
TFAM	5'-TGTGGGAGCTTCTCACTCTG-3'	5'-CTGGCCTCAAACCCATCCTA-3'
ACADM	5'-TAATTGGTGACGGAGCTGGT-3'	5'-TCCAGGGCATACTTGGTAGC-3'
RBFOX3	5'-CTCAGCAAAGCAGACCTCAC-3'	5'-TGGCAGAATTTCCTCTGGCT-3'
TUBB3	5'-GACCTACTGCATCGACAACG-3'	5'-CGATACCAGGTGGTTGAGGT-3'
MAP2	5'-CCAAGCAGCTTCGGCTTATT-3'	5'-CTGTGGCGGATGTTCTTCAG-3'
GAPDH	5'-ACAACTTTGGTATCGTGGAAGG-3'	5'-GCCATCACGCCACAGTTTC-3'

Supplementary Figure 1. Experimental schemes and primer lists of qRT-PCR. A. Experimental scheme to investigate the effect of exendin-4 (Ex-4) on expression levels of genes, proteins, ROS accumulation, and immunostaining under palmitic acid (PA)-induced apoptosis in SH-SY5Y cells treated with Vehicle (C or Ctr), Exendin-4 (E or Ex-4), Palmitic acid (P or PA) and both Palmitic acid and Exendin-4 (PE or PA+Ex-4). **B**. Experimental scheme to investigate the effect of Ex-4 on insulin resistance, mitochondrial dysfunction, and

neurite complexity under PA on expression levels of genes, proteins, immunostaining, neurite complexity in SH-SY5Y cells treated with reagents described in A. **C**. Experimental scheme to investigate the effect of Ex-4 on neurite complexity and immunostaining in primary cortical neurons DIV7 treated with reagents described in A. **D**. Experimental scheme to investigate the effect of Ex-4 on protein expression, synaptic plasticity, and dendritic spine morphology analysis in primary cortical neurons at DIV 21 treated with reagents described in A.

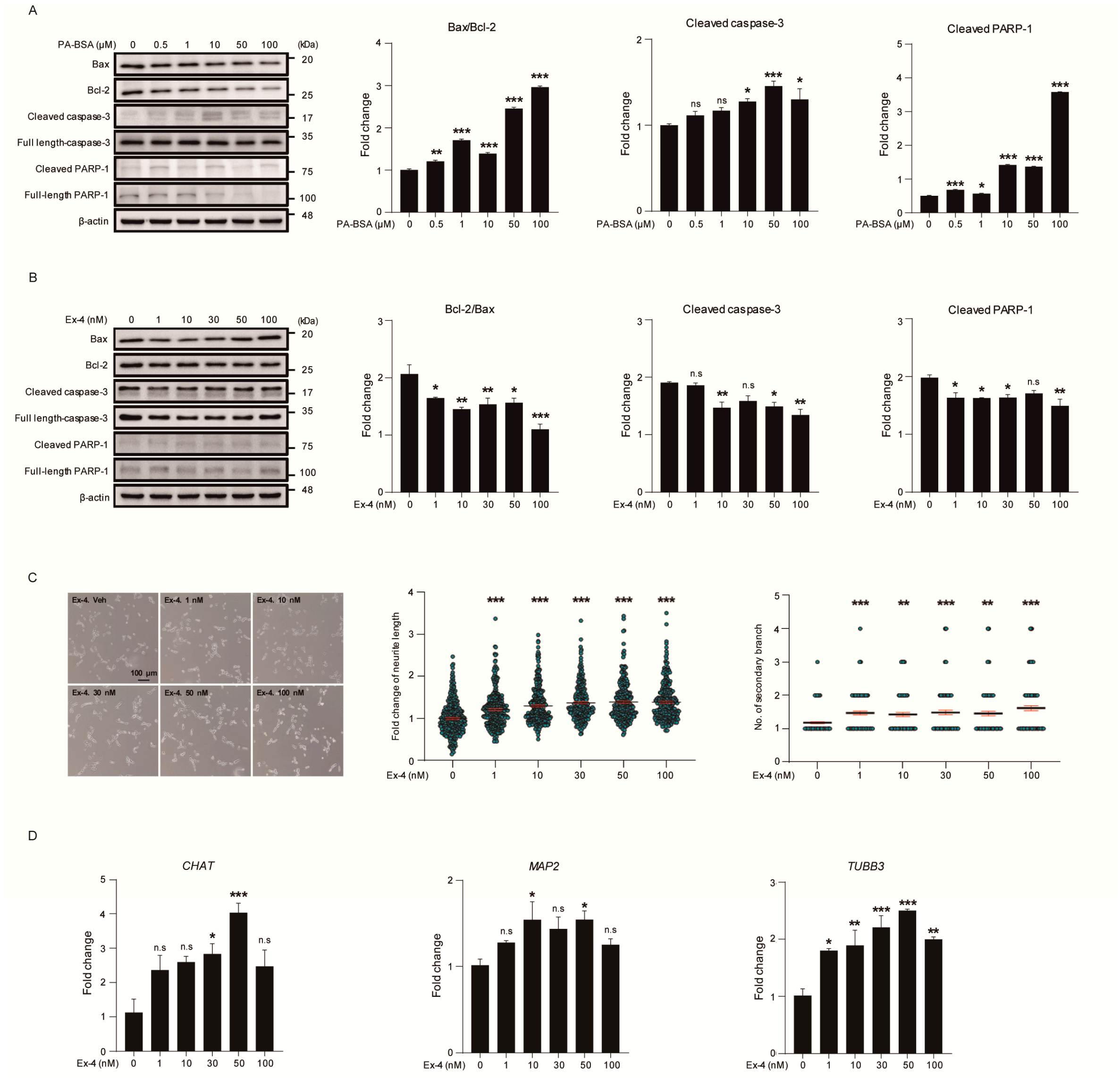

Supplementary Figure 2. Identification of palmitic acid and exendin-4 treat concentration in SH-SY5Y cells. **A.** PA-induced neuronal apoptosis signaling in SH-SY5Y cells treated with Vehicle (C or Ctr), Exendin-4 (E or Ex-4), Palmitic acid (P or PA), and both Palmitic acid and Exendin-4 (PE or PA + Ex-4). The protein level is normalized to β-actin. The cleaved-protein level of cleaved form is normalized to full-length form. **B.** Ex-4 suppressed PA-induced neural apoptosis in SH-SY5Y cells treated with reagents described in A. Each protein level is normalized to β-actin. The cleaved-protein level of cleaved form is normalized to full-length form. **C**. Ex-4 improves neural complexity (neurite outgrowth and the number of secondary branches) under PA-induced neuronal damage in SH-SY5Y treated with reagents described in A. Scale bar: 100 μm. **D**. Ex-4 improves RA-induced neuronal differentiation (*CHAT*, *MAP2*, and *TUBB3*) in SH-SY5Y cells treated with reagents described in A. The mRNA level of each gene is normalized to *GAPDH* level. Data information: In (A-D), error bars

represent S.E.M. *$p < 0.05$, **$p < 0.01$, ***$p < 0.001$ (Data A and B analyzed with ordinary one-way ANOVA. Data C and D analyzed with unpaired two-tail t-tests with Welch's correction).

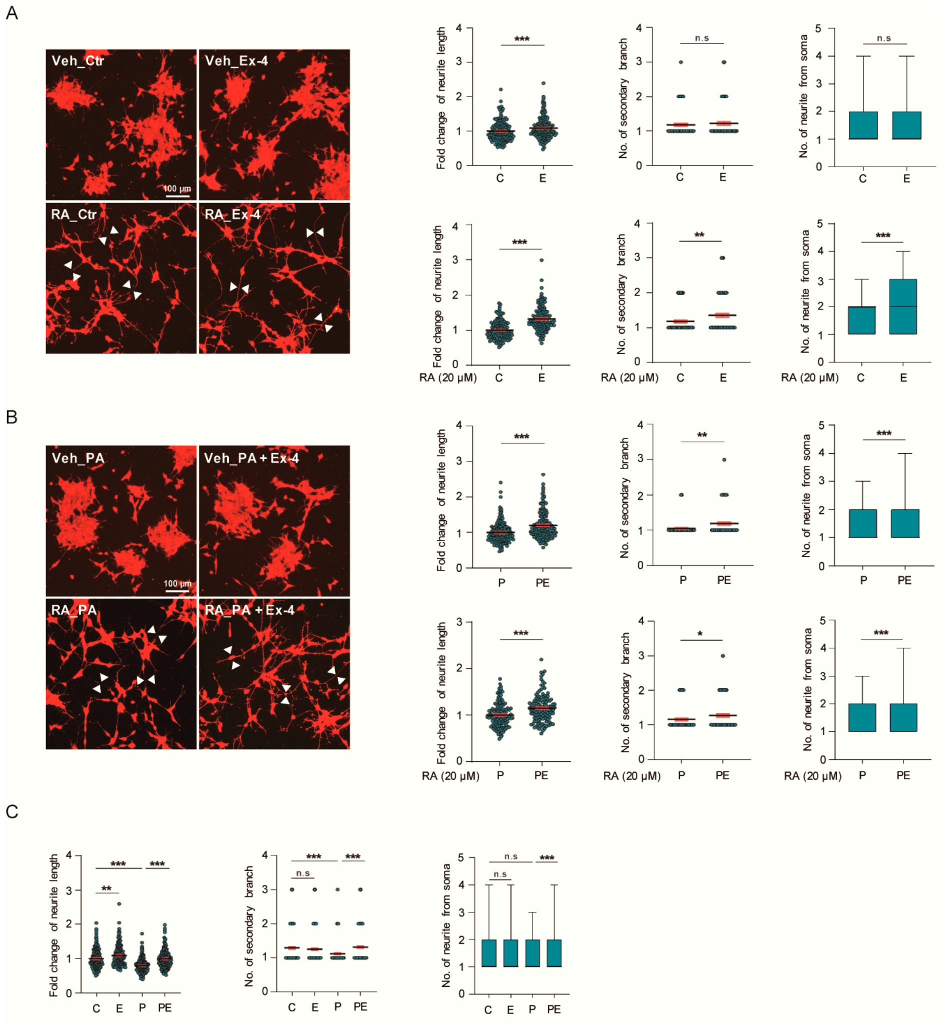

Supplementary Figure 3. Comparison of fluorescent images and histograms of undifferentiated- and differentiated SH-SY5Y cells. A. Comparison histograms of neurite complexity (neurite length, number of secondary branches, and neurite from soma) in veh cells and Ex-4 treated cells under undifferentiation and RA-induced differentiation condition. Scale bar: 100 μm. The white point indicates increased neurite site of the Ex-4 treated group compared with the veh group. **B**. Comparison histograms of neurite complexity (neurite length, number of secondary branches, and neurite from soma) in PA treated cells and PA + Ex-4 treated cells under undifferentiation and differentiation condition. Scale bar: 100 μm. The white point indicates PA-induced damaged neurite

site and recovered neurite of Ex-4 treated group. **C**. Comprehensive histograms in undifferentiated SH-SY5Y cells.

Data information: Error bars represent S.E.M. *$p < 0.05$, **$p < 0.01$, ***$p < 0.001$ (unpaired two-tail t-tests with Welch's correction).
